# The Effect of Dietary Synbiotics in Actively Racing Standardbred Horses Receiving Trimethoprim/Sulfadiazine

**DOI:** 10.3390/ani13142344

**Published:** 2023-07-18

**Authors:** Maria Lagounova, Jennifer L. MacNicol, J. Scott Weese, Wendy Pearson

**Affiliations:** 1 Department of Animal Biosciences, University of Guelph, Guelph, ON N1G 2W1, Canada; mlagouno@uoguelph.ca (M.L.); jmacnico@uoguelph.ca (J.L.M.); 2 Department of Pathobiology, University of Guelph, Guelph, ON N1G 2W1, Canada; jsweese@uoguelph.ca

**Keywords:** probiotics, synbiotics, fecal microbiome, horses, antibiotics

## Abstract

**Simple Summary:**

Synbiotics are often provided to horses receiving antibiotics to protect against disturbances of gut microbioma, despite a lack of evidence for efficacy. The purpose of this study was to evaluate the effect of a synbiotic (PROBIOPlus^TM^) in horses receiving antibiotics. Sixteen actively racing Standardbred horses were randomly allocated to one of four groups: antibiotics (10 days; AB), synbiotic (28 days; PBP), PBP + AB, or Control. Make-up of the bacterial gut population was assessed, as well as indicators of manure quality. The PBP + AB group had a significantly different population of bacteria in their manure compared with all other groups. Most of the differences were found in bacterial populations that function to degrade fiber, including *Fibrobacter* and *Ruminococcaceae*. The *Fibrobacter* population was significantly higher in AB and PBP + AB horses than Control. For *Ruminococcaceae*, Control was significantly higher than AB and PBP during antibiotic treatment, and PBP + AB horses were significantly higher than PBP horses after antibiotic treatment. In conclusion, these data provide support for the ability of PROBIOPlus™ to maintain healthy gastrointestinal microbiome during antibiotic treatment.

**Abstract:**

Synbiotics are often provided to horses receiving antibiotics to protect against microbiome disturbances, despite a lack of evidence for efficacy. The purpose of this study was to evaluate the effect of a synbiotic product in horses receiving antibiotics. Sixteen actively racing Standardbred horses were randomly allocated (four-way crossover) to one of four groups: antibiotics (10 days; AB), synbiotics (28 days; PROBIOPlus^TM^; PBP), PBP + AB, or Control. The fecal microbiome was investigated using 16S rRNA sequencing, and fecal dry matter (DM; %), pH, and scores (FS; 0–9) were measured. Data were analyzed with two-way ANOVA. Results found microbiota differences in community membership between PBP + AB and all other treatments during and after antibiotic treatment. During antibiotic treatment, AB and PBP + AB were significantly different from Control. After antibiotic treatment, PBP + AB was significantly different from all other treatments. The few differences found in relative abundance of phyla or predominant genera were mostly in fiber degrading bacteria. The Fibrobacter population was significantly higher in AB and PBP + AB horses than Control. Unclassified *Ruminococcaceae* was significantly higher in Control than AB and PBP. After antibiotic treatment, PBP + AB horses were significantly higher than PBP horses. In conclusion, these data provide support for the ability of PROBIOPlus™ to maintain healthy gastrointestinal microbiome during antibiotic treatment.

## 1. Introduction

Antimicrobials are commonly used in equine clinical practice. Antibiotics can benefit equine patients by reducing infection, recovery time, mortality, and loss of performance [1]. However, antibiotic use can also result in significant and severe side effects. Seizures, neurological effects, nephrotoxicity, dysrhythmia, and severe diarrhea or colitis are some of the reported adverse reactions to antibiotic use in horses [2]. Furthermore, all classes of clinically relevant antibiotics have been documented to cause antimicrobial associated diarrhea (AAD) in horses [3]. The etiology of AAD is still relatively unknown, and likely there are a combination of contributing factors. Nevertheless, a disturbance in the normal gastrointestinal (GI) microflora caused by antibiotic treatment is generally accepted as a major contributing factor. It has been postulated that the disturbance in the normal GI microbiota caused by antibiotic therapy results in a loss of colonization resistance to pathogens, allowing the proliferation of pathogenic bacteria and production of toxins. Baveurd et al. [4] identified *C. difficile* or its toxin in 40% of horses that developed colitis when treated with antibiotics for a condition other than diarrhea. Interestingly, all horses that developed colitis in this study, regardless of whether *C. difficile* was identified, were treated with a β-lactam class antibiotic or β-lactam in combination with another antibiotic. The methods to protect against the loss of commensal bacteria and subsequent GI colonization with opportunistic pathogens during antibiotic treatment represent an important area of study.

Enteric biologics, including probiotics (direct-fed microbials), prebiotics (microbial growth substrate which is indigestible by the horse), and synbiotics (combination of pro- and prebiotics), are popular in the nutritional supplement industries due to their perceived natural health benefits and general safety. Most research pertaining to enteric biologics has focused on probiotics. The Food and Agricultural Organization of the World Health Organization define probiotics as “live microorganisms which, when administered in adequate amounts, confer a health benefit on the host” [5]. Mechanisms of action of probiotics include antimicrobial production, immune enhancement, bacterial toxin inactivation, and competitive exclusion of pathogenic bacteria [6,7]. In humans, the use of probiotics to prevent and treat AAD has been explored with some promising results. The probiotic bacteria *Lactobacillus* GG was found to reduce the incidence of AAD in children treated with oral antibiotics [8]. The yeast *Saccharomyces boulardii* given for 2 days prophylactically and throughout a course of antibiotic treatment significantly reduced the incidence of AAD in patients treated with β-lactams [9]. The greater reliance of horses upon a robust GI microbial environment when compared to humans makes a strong case for the potential of probiotics to combat AAD in equines.

In horses, the use of probiotics to maintain a normal GI microflora during dietary challenges has been explored. Jouany et al. [10] fed the yeast *Saccharomyces cerevisiae* to cannulated horses on either high-fiber or high-starch diets. The yeast was not found in GI contents of unsupplemented individuals but was recovered from cecal and colonic contents of supplemented animals indicating its survival through the GIT. Furthermore, increases in lactobacilli and lactic acid utilizing bacteria were evident in the cecum of supplemented horses. These alterations in the distal GI microbiota, along with increases in the activity of fiber-degrading enzymes, support previous results that demonstrate improved fiber digestion in horses supplemented with *S. cerevisiae*. Conversely, supplementing horses with either *Lactobacillus acidophilus* or a probiotic combination of *L. acidophilus*, *L. casei*, *Bifidobacterium bifidum*, and *Enterococcus faecium* did not have any effect on nutrient digestibility or reducing the risk of acidosis [11]. Clearly, not all probiotic bacteria confer a measurable effect on GI health when fed to horses. The efficacy of commercially manufactured probiotic supplements cannot be assumed; each product must be tested within the bounds of its expected use and performance.

PROBIOPlus^TM^ (PBP; Selected Bioproducts Inc., Guelph, ON, Canada) is a commercially available enteric biologic which is labelled to contain probiotic organisms (*Bacillus coagulans*, *Bifidobacterium animalis*, *Enterococcus faecium*, and *Lactobacillus acidophilus*), prebiotic substances (chicory, inulin, and fibres from flax and fenugreek), and digestive enzymes (amylase, protease, glucanase, and xylanase). We previously explored the effect of PBP on metabolism and growth of equine cecal microorganisms grown in a chemostat or anaerobe chamber [12]. In this study, a consistent stimulatory effect of PBP on the metabolic profile of cultures in both culture methods was observed, evidenced by higher production of acetate, propionate, and butyrate. These data support the application of PBP as an effective modulator of gastrointestinal microbiome metabolism, primarily through a prebiotic effect. What is not known, however, is whether this effect is reproducible in the live horse or whether it will normalize the enteric microbiome in the face of a physiological challenge. Thus, the objective of the current study was to compare the effects of PBP with that of a common oral antibiotic (A; Trimethoprim/Sulfadiazine), their combination (PBP + A), and an unsupplemented control diet (CO) on fecal microbial and physical characteristics in actively racing Standardbred horses.

## 2. Materials and Methods

This study was conducted at a private Standardbred racing facility in Puslinch, Ontario, Canada. All experimental procedures and conduct were approved by the University of Guelph Animal Care Committee under the standards of the Canadian Council of Animal Care (AUP#4205). Signed informed consent was obtained by horse owners prior to enrollment in the study.

### 2.1. Animals

Adult Standardbred horses (*n* = 16) in active race training were recruited into this study. Mares, stallions, and geldings with ages ranging from 2 to 7 year (median 3 year) and weights ranging from 460 to 567 kg (mean 502.3 kg) were enrolled. Horse characteristics are detailed in Table 1.

All horses received a standard diet that consisted of mixed timothy-alfalfa hay (1.5% of body weight), 7 kg Brooks Leading Edge Complete Feed (Brooks Equine Nutrition, Guelph, ON, Canada), 0.7 kg of beet pulp pellets, and 0.33 kg Peak Performance Nutrients Mineralade (Peak Performance Nutrients, Delray Beach, FL, USA) daily. Water was provided ad lib.

Horses were exercised 6 days per week (Monday–Saturday) beginning at 5 a.m. The daily exercise routine consisted of 20–25 min of jogging under tack. Horses also underwent 1 (if horse was scheduled to race that week) or 2 (if horse was not scheduled to race that week) simulated racing work outs, in addition to their daily exercise. After training, horses were turned out in small turnout paddocks until approximately 1 p.m., after which time they were returned to their stalls (10′ × 12′) until the next day.

Weekly general health assessments were performed on all horses during the trials, which included rectal temperature, heart rate (stethoscope), respiratory rate, hydration status (skin pinch), and 4 quadrant gut sounds. Fecal scoring was performed on days −1, 0, 1, 2, 9, 10, 11, 28, 29, and 30 to monitor for occurrence of AAD. A fecal score of 1 (dry balled)–9 (yellow diarrhea) adapted from John et al. [13] was assigned to freshly voided feces based on visual assessment.

### 2.2. Experimental Design

**Figure 1:**
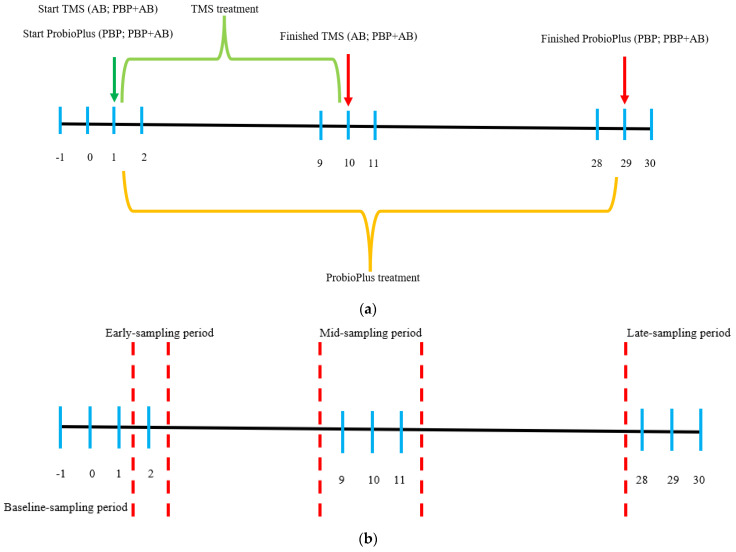
(**a**). Depiction of treatment durations and sampling days during each trial. Light blue lines represent fecal sampling days. Green arrow indicates the beginning of TMS and PROBIOPlus treatment. Red arrows represent the end of treatments. Green bracket represents TMS treatment period. Orange bracket represents PROBIOPlus treatment period. (**b**). Depiction of sampling periods and sampling days during one trial. Light blue lines represent fecal sampling days. Red dashed lines represent divisions between sampling periods.

Four treatments were investigated in this experiment, which was designed as a 4-way crossover with a total of 4 trials completed over the period of November 2019–June 2020. In each of the 4 trials, 8 horses (2 per treatment) were utilized. Each trial was a total of 32 days in length (day −1–day 30) with a 28-day washout period between trials. Horses received one of each of the supplement in each trial, resulting in a final ‘n’ of 8 horses per treatment group. Due to COVID-19, Standardbred racing was temporarily shut down during trial 3. A visual representation of the experiment and sampling time course is presented in Figure 1a.

The 4 treatments were:

- Antibiotic only (AB; Trimethoprim/Sulfadiazine, 75 mg/kg BW administered by oral syringe once per day on days 1–10).
- Synbiotic only (PBP; PROBIOPlus^TM^ (Selected Bioproducts Inc., Guelph, ON, Canada), 30 mg/kg BW administered as a feed top-dress once per day (per manufacturer directions) on days 1–28).
- Synbiotic + Antibiotic (PBP + AB).
- Unsupplemented control (CO).

Not all horses were able to complete all 4 trials (and therefore all 4 treatments) as some were sold or returned to their home farm. If a horse could not complete the entire experiment, it was replaced by a different horse to complete the trials and treatments. Therefore, a complete crossover design could not be accomplished. The treatments and trials completed by each horse enrolled are included in Table 1.

### 2.3. Sampling

Fecal samples were taken on trial days −1, 0, 1, 2, 9, 10, 11, 28, 29, and 30. Visual assessment for fecal scoring [13] was performed on each sample prior to placing them into sterile cups. Cups were immediately stored in a cooler on ice until all samples had been collected. They were then transported in the cooler to the University of Guelph and placed into a −80 °C freezer until analysis.

### 2.4. DNA Extraction and Illumina Sequencing

Fecal samples were thawed overnight in a refrigerator. DNA extraction was performed using a standard kit (ENZA Stool DNA Kit, Omega BioTek, Norcross, GA, USA) according to the manufacturer’s instructions. Extracted DNA was stored at −20 °C until PCR sequencing could be performed. The V_4_ region of the 16S rRNA gene was amplified using modified primers [14] and KAPA2G Fast Hot Start Ready Mix (Sigma Aldrich, Oakville, ON, Canada). PCR products were then visualized on a 1.5% agarose gel and purified using MagBind magnetic beads (Omega BioTek, Norcross, GA, USA). Following visual confirmation, a second PCR was performed using Illumina SetD Indexing primers (Animal Health Laboratory, University of Guelph, Guelph, ON, Canada). Samples were again purified and then sent to be sequenced on an Illumina Miseq (Agriculture and AgriFood Lab, University of Guelph, Guelph, ON, Canada). A total of 320 samples were sent for sequencing.

Bioinformatics analysis was performed using the opensource software mothur [15,16] v1.34. Pair end reads were aligned and underwent a standard series of quality control filtering steps [17]. Alpha diversity indices including richness (the number of taxa present within a sample), evenness (the relative abundance of the taxa within a sample), and diversity (accounts for both richness and evenness) were calculated within mothur. Beta diversity measures of community membership (taxa that are not present within a community of samples) and community structure (accounts for membership as well as abundance) were also calculated using mothur. Structure and membership were visualized using PCOA plots. Community characteristics were compared using AMOVA (analysis of molecular variance) and HOMOVA (homogeneity of molecular variance). Linear discriminant analysis effect size (LEfSe) was used to identify OTUs that were differentially abundant between groups.

### 2.5. Bioinformatics

To simplify comparisons within mothur, days −1, 0, 1, and 2 were compared; days 9, 10, and 11 were compared; and days 28, 29, and 30 were compared. As no differences were noted with these comparisons, samples from these days were then grouped into periods as follows:

Baseline period: days −1, 0, and 1 (prior to treatment)
Early period: day 2 (commencement of treatment)
Mid period: days 9, 10, and 11 (end of AB treatment)
Late period: days 28, 29, and 30 (end of PBP treatment)

A visual representation of the grouping of sampling days is presented in Figure 1b.

### 2.6. Fecal pH and Dry Matter Measurements

Fecal samples were thawed overnight, and pH was measured using a Sartorius™ pH basic meter (Sartorius AG, Niedersachsen, Germany). The probe was placed directly into the fecal sample to measure pH. The probe was rinsed with deionized water and dried between measurements.

Following pH measurements, approximately 20 g (wet weight) of fecal material was removed from the container and placed on a 10 × 10 cm^2^ sheet of tinfoil; the remainder of the fecal sample was returned to the −80 °C freezer. The tinfoil sheet was placed into a drying oven (60 °C) for 72 h. After drying, samples were removed from the oven and weighed (dry weight). Dry matter (%) was calculated as (dry weight/wet weight) × 100.

### 2.7. Synbiotic Culture

Five lots of manufactured product were used over the course of the trial. A representative sample of the product from each lot was collected directly from a commercial supplement tub. A sample of the lyophilized base probiotic mix that was used for supplement manufacturing was also collected. Samples were placed in a −80 °C freezer until further processing.

Samples from all lots were cultured aerobically on phenylethyl alcohol agar (Oxoid Limited, Nepean, ON, Canada), as well as anaerobically on Muller Hinton agar (Sigma Aldrich, Oakville, ON, Canada). One (1.0) g of sample was serially diluted in 9 mL of sterile PBS and plated in triplicate from a concentration of 10^−1^–10^−9^. Aerobic plates were cultured for 24 h at 37 °C, and anaerobic plates were cultured in a DG250 Whitley anaerobic chamber (Microbiology International, Frederick, MD, USA) at 37 °C for 48 h. Plates with <300 colonies were counted, and the average between all 3 plates was used to determine viable bacteria. Visual assessment of morphology was used to differentiate bacteria, and representative colonies were sub-cultured and sent to Animal Health Laboratory (University of Guelph, Guelph, ON, Canada) for MALDI-TOF identification.

### 2.8. Statistics

Statistical analyses were performed in SAS v.9.4 using PROC GLIMMIX. The optimal model run in ANOVA was based on the assessment of residuals. Data are presented as means ± SEM, and *p* < 0.05 was considered significant.

Alpha diversity, including Chao1 (richness), Shannon Inverse Evenness Index (evenness), and Inverse Simpson Index (diversity), taxonomy, fecal dry matter, and fecal pH were assessed via RM ANOVA according to the following model:

γ_ijk_ = μ + treatment_i_ + period_j_ + treatment × period_ij_ + treatment × day(period)_k_ + horse_l_ + ε_ijkl_

where μ is the overall mean, treatment is the effect of the treatment (i = PBP, AB, PBP + AB, CO), period is the effect of the predefined sampling periods of grouped days (j = baseline, early, mid, and late), treatment × period is the interaction between the treatment and sampling periods, treatment × day (period) is the nested effect of the individual sampling days within their sampling period for each treatment, horse is the random effect of horse, and ε_ijk_ is the residual error. Post hoc comparisons were adjusted using a Tukey–Kramer adjustment. Phyla had a total relative abundance of greater than 1%, and genera had a total relative abundance of greater than 2%. The Benjamini–Hochberg procedure was used to control the False Discovery Rate (FDR) for taxonomic comparisons using sequential modified Bonferroni correction for multiple hypothesis testing in taxonomic comparisons. A *p*-value of ≤0.05 was considered significant for all comparisons.

## 3. Results

### 3.1. Microbiome Analysis

A total of 37,945,565 sequences passed through quality control filtering. Sequences per sample ranged from 45,946 to 391,787 (median 117,931). Subsampling at a depth of 40,000 sequences per sample was performed to normalize sequences across sampling [18].

### 3.2. Beta Diversity

When treatments at each sampling period were compared, no differences in community structure (Yue and Clayton index) or membership (Jaccard index) as assessed by AMOVA or HOMOVA were found between any of the sampling periods in PBP, AB, or CO. Community membership, when assessed by AMOVA, demonstrated a difference between PBP + AB in the mid- and late sampling periods. Furthermore, during the mid-sampling period CO significantly differed from AB (*p* = 0.012), PBP + AB (*p* = 0.002), and PBP (*p* = 0.05). PBP was also significantly different from AB (*p* = 0.001) and PBP + AB (*p* < 0.001) during the mid-sampling period. In the late sampling period, there was a significant difference between PBP + AB and CO (*p* = 0.004), AB (*p* = 0.003), and PBP (*p* < 0.001). However, no distinct clustering could be visualized on PCOA plots.

### 3.3. Alpha Diversity

No significant differences in richness were noted. A significant interaction between treatment and sampling period was evident in evenness (*p* = 0.043, Table 2). There was also a significant effect of period (*p* = 0.041; Table 3) and interaction between sampling period and treatment in diversity (*p* = 0.0071; Table 3). Furthermore, in the early sampling period PBP had significantly lower diversity than CO (*p* = 0.026; Table 3).

### 3.4. Taxonomy

There were seven phyla with a total relative abundance of greater than 1%. These included Firmicutes (45.3%), Bacteroidetes (25.3%), Verrucomicrobia (8.7%), Spirochaetes (2.2%), Fibrobacteres (2.1%), and Actinobacteria (1.3%).

**Figure 2:**
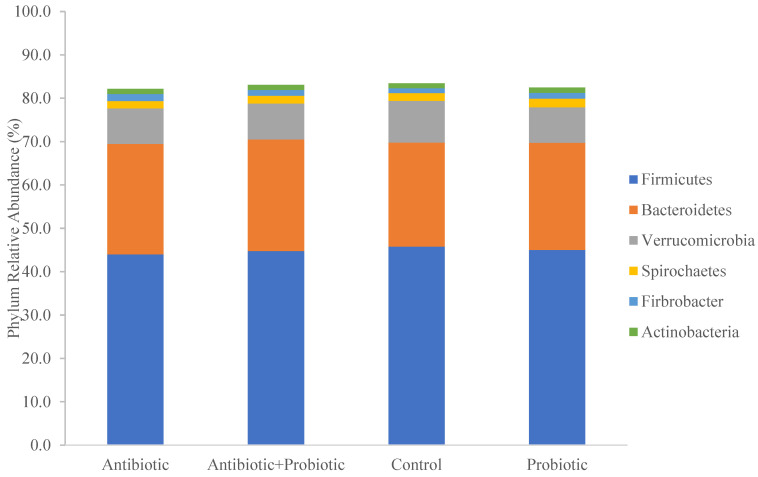
Relative abundances of the top six phyla by treatment. Eight (8) horses were treated with either no antibiotic or synbiotic (CO), 10 days with oral antibiotic (AB; Trimethoprim/Sulfadiazine; 75 mg/kg BW/day), 28 days with a top dressed commercial synbiotic supplement (PBP; PROBIOPlus^TM^, Selected BioProducts Inc., Guelph, ON, Canada; 30 mg/kg BW/day as per the manufacturer’s instructions), or both AB for 10 days and PBP for 28 days (PBP + AB).

There was a significant effect of treatment on the relative abundance of Bacteroidetes (*p* = 0.02; Figure 2). The population of Bacteroidetes was lower in CO (24.0 ± 0.006%) compared to PBP + AB (25.7 ± 0.006%; adj. *p* = 0.026).

There were nine genera with a total relative abundance of greater than 2%. These included an unclassified member of Bacteroidetes (17.2%), an unclassified member of Ruminococcaceae (11.6%), an unclassified member of Lachnospiraceae (11.5%), an unclassified member of Clostridiales (6.4%), an unclassified member of Subdivision 5 (5.8%), an unclassified member of Bacteroidales (4.6%), an unclassified member of Firmicutes (3.8%), *Trepomona* (2.1%), and *Fibrobacter* (2.1%).

There was a significant effect of treatment on the relative abundance of unclassified Bacteroidetes (*p* = 0.035), *Fibrobacter* (*p* = 0.0024), and unclassified *Ruminococcaceae* (*p* = 0.0037).

The relative abundance of *Fibrobacter* was significantly higher in AB (1.66 ± 0.003%) than CO (1.14 ± 0.002%; adj. *p* = 0.0012) and PBP (1.31 ± 0.002%; adj. *p* = 0.045). The relative abundance of unclassified *Ruminococcaceae* was significantly higher in CO (12.5 ± 0.004%) than AB (11.3 ± 0.004%; adj. *p* = 0.0066) and PBP (11.1 ± 0.004%; adj. *p* = 0.006). In the late sampling period, unclassified *Ruminococcaceae* was lower in PBP (10.7 ± 0.005%) than either the CO (12.9 ± 0.006%; adj. *p* = 0.036) or PBP + AB (12.8 ± 0.006%; *p* = 0.042).

### 3.5. LEfSe Analysis

**Figure 3:**
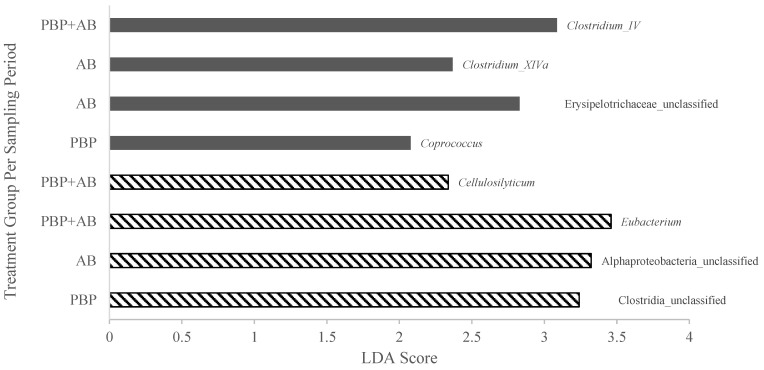
Enriched bacterial genera as assessed by LEfSe analysis with LDA scores greater than two per treatment during the mid-sampling period (corresponds to sampling days 9, 10, and 11; gray bars) and during the late sampling period (corresponds to sampling days 28, 29, and 30; dashed bars). Eight (8) horses were treated with either no antibiotic or synbiotic (CO), 10 days with oral antibiotic (AB; Trimethoprim/Sulfadiazine; 75 mg/kg BW/day), 28 days with a top dressed commercial synbiotic supplement (PBP; PROBIOPlus^TM^, Selected BioProducts Inc., Guelph, ON, Canada; 30 mg/kg BW/day as per the manufacturer’s instructions), or both AB for 10 days and PBP for 28 days (PBP + AB).

A total of seven OTUs from the phylum Firmicutes and one from Proteobacteria were differentially enriched within particular treatments during different sampling periods (Figure 3). In the mid-sampling period, *Clostridium* XIVa was enriched in AB; *Clostridium* IV was enriched in PBP; unclassified members of *Erysipelotrichaceae* and *Coprococcus* were enriched in the PBP + AB group. In the late sampling period, both *Eubacterium* and an unclassified member of *Alphaproteobacteria* were enriched in AB; *Cellulosilyticum* was enriched in PBP; and an unclassified member of *Clostridium* was enriched in PBP + AB.

### 3.6. Fecal pH and Dry Matter

**Figure 4:**
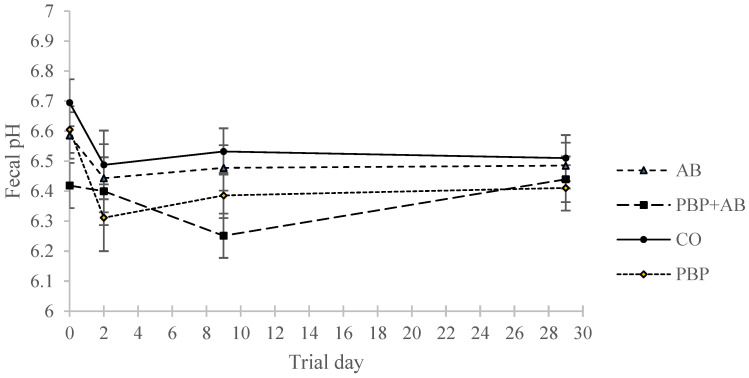
Fecal pH as measured by Sartorius™ pHbasic meter. Measurements correspond to the baseline sampling period (days −1, 0, and 1), early sampling period (day 2), mid-sampling period (days 9, 10, and 11), and late sampling period (days 28, 29, and 30). Eight (8) horses were treated with either no antibiotic or synbiotic (CO), 10 days with oral antimicrobial (AB; Trimethoprim/Sulfadiazine; 75 mg/kg BW/day), 28 days with a top dressed commercial synbiotic supplement (PBP; PROBIOPlus^TM^, Selected BioProducts Inc., Guelph, ON, Canada; 30 mg/kg BW/day as per the manufacturer’s instructions), or both AB for 10 days and PBP for 28 days (PBP + AB).

There was a significant effect of treatment (*p* = 0.017) and sampling period (*p* = 0.0008) on fecal pH. PBP + AB had a significantly lower pH (6.34 ± 0.06) compared to CO (6.55 ± 0.06, *p* = 0.02; Figure 4). When pH was assessed during each sampling period between treatments, it was numerically lowest in PBP + AB during the mid-sampling period (6.25 ± 0.07). However, the difference in pH did not reach statistical significance between the treatments during this sampling period, and fecal pH remained within reference intervals reported for healthy horses throughout the study [19]. No differences were noted in fecal DM. All fecal samples during the experiment were within the normal range between a score of 2 and 3.

### 3.7. Synbiotic Culture

According to the manufacturer’s label, PROBIOPlus^TM^ contains four added probiotic bacteria with a total 2.2 × 10^11^ CFU/g. These probiotic bacteria include *Bacillus coagulans*, *Bifidobacterium animalis*, *Enterococcous faecium*, and *Lactobacillus acidophilus.* Bacterial strain information is not provided. Bacterial colonies confirmed by MALDI-TOF analysis on culture plates from each LOT included *Pediococcous acidilactici* and *Enterococcous faecium.* In the probiotic base, *P. acidilactici* had a count of 1.7 × 10^11^ CFU/g, and *E. faecium* had a count of 3.4 × 10^10^ CFU/g. Across all five LOTs, total bacterial counts ranged from 3.1 × 10^6^ to 2.5 × 10^7^ CFU/g (average 1.3 × 10^7^ CFU/g/LOT). Counts of *P. acidilactici* ranged from 9 × 10^5^ to 9.7 × 10^6^ (average 3.9 × 10^6^ CFU/g/LOT). Counts of *E. faecium* ranged from 2.2 × 10^6^ to 1.5 × 10^7^ (9 × 10^6^ CFU/g/LOT).

## 4. Discussion

Antibiotics are an important medical treatment and are required to combat bacterial infections in certain cases. However, commensal communities of bacteria are integral for host health. This is particularly relevant within the GIT as a large part of its function is dependent upon the highly complex microbiota that resides therein. Many antibiotics, particularly broad-spectrum antibiotics, indiscriminately act against all bacteria resulting in the loss of necessary microbes and an overall decrease in GI microbial diversity [20,21]. A loss of microbial diversity is generally considered problematic and is often associated with GI disease. Some examples of this include ulcerative colitis, Crohn’s disease, and *C. difficile* AAD in humans which are all accompanied by a decrease in GI microbial diversity [22,23]. Reduced microbial diversity has also been noted in horses suffering from post-partum colic [24] and those presenting at a referral practice with a diagnosis of colic [25]. However, reduced microbial diversity is not a universal finding during disease, and, conversely, reduced microbial diversity can be observed in clinical healthy individuals [26]. As such, care must be taken to remember these data do not prove causation but rather they suggest that decreased microbial diversity may play a role in increased risk for gastrointestinal disease.

In this study Firmicutes, Bacteroidetes, and Verrucomicrobia were the predominant phyla in equine feces. These results agree with other reports on the equine fecal microbiome [27,28,29]. Although these represent the most prevalent phyla in many studies, the relative abundances are inconsistent. These discrepancies, at least in part, are likely due to differences in the method of sample collection, sample storage conditions, and laboratory techniques as these factors can impact sequencing output [30,31,32,33]. Furthermore, the use of different bioinformatics pipelines and databases can influence taxonomy results.

All the horses given oral AB during this study remained clinically healthy, and this is consistent with others who report no clinically significant effect of TMS-associated decline in microbiome diversity [26]. No negative effects of any treatments were noted during weekly health assessments, and no evidence of diarrhea was observed during fecal scoring. This was expected and is consistent with the relatively frequent use of this AB in equine veterinary practice. Nevertheless, the microbial profile of horses with and without AAD is similar, with the only distinguishing taxonomic difference being in Verrucomicrobia [34]. Therefore, microbial alterations seen in horses on antibiotics without diarrhea are still likely have a degree of relevance when considering clinical cases of AAD.

Although there was minimal evidence of significant microbial community shifts due to the treatments used in this study when evaluating alpha and beta diversity measures, some interesting taxonomic differences were noted in horses treated with AB or a combination of PBP + AB. In particular, the LEfSe analysis identified OTUs that were differentially enriched in the mid- and late sampling periods within the treatment groups. These alterations in particular taxa may have important implications on how the equine GIT responds to external pressures. Following 9 days of antibiotic treatment an enhancement in the bacterial group, *Clostidium* XIVa was evident. This is somewhat unexpected as *Clostridium* XIVA is generally considered beneficial for gut health. It is a butyrate producer along with *Clostridium* IV [35,36], which was the bacterial cluster enriched during the mid-sampling period in PBP horses. These *Clostridium* clusters exert anti-inflammatory effects and have been considered as potential probiotics [34]. Furthermore, the loss of *Clostridium* XIVa and *Clostridium* IV has been associated with the loss of colonization resistance 72 h following the use of broad-spectrum antibiotics in patients admitted to the ICU [37]. Therefore, it is interesting that they were upregulated in both the AB and PBP treatments in this study. Additionally, direct comparisons of taxonomic relative abundances demonstrated a significantly higher relative abundance of the genus *Fibrobacter* in both groups of horses that received antibiotic treatment. *Fibrobacter* strains residing in hindgut fermenting hosts are diverse but demonstrate a conserved specialization in cellulose fermentation [38]. The PBP + AB treatment group also exhibited an enrichment of *Coproccocus*, another butyrate producing bacteria [39] during the mid-sampling period. *Coproccocus* has been postulated to support gut health by synthesizing acetate and b-vitamins [40]. It is interesting that what are generally considered beneficial bacteria are enhanced in feces from horses following 9 days of treatment with antibiotics. Collinet et al. [41] observed a similar event wherein the genera *Lachnoclostridium* and *Ruminococcaceae* were discriminating features in the microbiome of horses during TMS treatment. The authors postulated that these microbial changes might represent an adaptive response to maintain the fiber degrading ability during TMS induced dysbiosis.

During the late sampling period, the results of microbial alterations between treatments are less clear. This is primarily due to the unclassified nature of the genera that are enriched or the lack of information regarding the specific genera which were altered. In the antibiotic treatment, *Eubacterium* species were enriched. *Eubacterium* species are a normal component of the GI microflora and are of fairly limited clinical importance [42]. In the probiotic treatment group, *Cellulosilyticum* species were enriched. A strain of this bacterium, isolated from the rumen of yak, possesses specific fiber degrading strategies that may be particularly relevant for animals that subsist on forage rich diets [43]. The fibrolytic enzyme production was specifically ascribed to the *Cellulosilyticum ruminicola* H1 strain [43]. Unfortunately, a limitation of 16S variable region short-read sequencing is the lack of certainty regarding species level resolution [44]. Strain resolution was beyond our capabilities using this methodology. Furthermore, to the authors’ knowledge *Cellulosilyticum ruminicola* has not been identified in equine fecal samples. Thus, attributing an improvement in the specific fiber degrading capacity of the H1 strain following PBP treatment requires further research. A hypothesis of improvement in fiber degrading capacity afforded by enrichment of *Cellulosilyticum* following 28 days of probiotic supplement may be challenged by the concurrent lower relative abundance of unclassified *Ruminococcaceae* observed in this group at the late sampling period when compared to controls. Although the genus was unclassified, the family *Ruminococcaceae* are also a group of fiber-degrading butyrate producers [45]. Thus, the reduction in *Ruminococcaceae* during the late sampling period appears to represent a functional conflict with the enrichment of *Cellulosilyticum.* Culture based work to explore and isolate relevant microbes and specifically investigate their fermentative/metabolic capacities could aide in illuminating the practical relevance of these results.

Fecal pH was numerically lower during the mid-sampling period in PBP + AB horses when compared to the other treatment groups. As the pH remained within a physiologically normal range and no adverse effects on health were noted, it is unlikely this change in fecal pH was linked to any type of GI metabolic acidosis due to an over production of lactic acid. *C. difficile* growth, sporulation, and toxin production are affected over a physiological range of pH and are reduced at more acidic pH [46]. Due to the importance of this particular pathogen with regard to AAD in equines, it would be interesting to explore this effect of PBP further. In vitro culturing could be a particularly useful and cost-effective tool to pursue this avenue of inquiry. At the moment, it is difficult to assess the relevance of this result, because no changes in lactic acid producing bacteria were identified, and therefore the source of the reduced fecal pH is not obvious. When PBP was previously assessed in vitro [12], its addition significantly increased SCFA production with a concomitant reduction in system pH. This may provide some insight into the mechanism for lower fecal pH in the current study, but this must be evaluated in future studies which quantify fecal metabolites. The addition of fecal metabolite analysis in the current study may have assisted in creating a context for the pH results that were observed.

There are several limitations which must be addressed when interpreting the results from this study. An *n* value of eight horses per treatment group is a fair size when performing live animal trials with horses. However, a true crossover design in which each horse was administered all treatments would have been ideal due to high inter-individual differences within the equine GI microbiota [47,48]. Although utilizing a crossover in which there was no subject attrition would have been ideal, the prolonged period the experiment required still represents a drawback in study design. Salem et al. [29] identified changes within the fecal microbiomes of horses managed on pasture over 12 months. Similarly, Blackmore et al. [47] identified intra-individual differences in fecal microbial characteristics as identified by DGGE from ponies taken with an 11-week interval. Nevertheless, these limitations increase variability and thus are more likely to obstruct potential differences between treatments. Therefore, the results from this study are likely to be on the more conservative side. The results from this study should be interpreted within the context of the study limitations. Nevertheless, these results do provide preliminary data regarding the adaptive response of equine GI microbial communities to oral antibiotic treatment with TMS and the concurrent administration of a synbiotic supplement.

The synbiotic used over the course of this study is a commercially manufactured product and represents a suitable example of a common supplement the typical horse owner would feed. The lack of specificity and consistency between the label and product is a longstanding issue within the probiotic supplement industry [49,50,51]. The lack of strain identification is particularly problematic as this precludes the determination of whether the probiotic bacterial strains used within the study were resistant to TMS. This resistance would be a requirement if an effect attributable to the probiotic bacteria were to be observed during concomitant TMS administration. Strain identification is also increasingly relevant as the identification of probiotic strains with transferrable antibiotic resistance genes challenges the commonly accepted view regarding the general safety of probiotics [52].

## 5. Conclusions

No direct effects of the synbiotic supplement used in this study on the equine fecal microbiome were obvious from these results. However, the enrichment of *Clostridium* XIVa and IV during treatment with the antibiotic and treatment with the synbiotic supplement demonstrates the potential of the equine gut to adapt to external pressures through microbial community shifts which might improve colonization resistance and mucosal health. There were also upregulations in fiber degrading bacterial taxa following the cessation of antibiotic treatment which could potentially represent an adaptive response to maintain microbial community activity and enhance recovery from antibiotic treatment.

## Figures and Tables

**Table 1:** Study horse demographics.

Horse	Sex	Age (yr)	Height (hh)	Weight (kg)	Treatment			
					Trial 1	Trial 2	Trial 3	Trial 4
1	G	3	15.2	485	CO	×	×	×
2	M	2	15.3	567	CO	PBP + AB	AB	PBP
3	M	2	15.3	540	PBP	CO	PBP + AB	AB
4	G	2	15.3	485	PBP	CO	AB	PBP + AB
5	S	2	15.3	460	AB	PBP	CO	PBP + AB
6	G	7	15.3	522	AB	PBP	×	×
7	G	6	15.2	472	PBP + AB	AB	×	×
8	G	4	16.2	540	PBP + AB	×	×	×
9	G	3	15.3	512	×	PBP + AB	×	×
10	S	2	15.1	512	×	AB	PBP	CO
11	G	2	15.1	460	×	×	PBP	×
12	M	3	16.2	540	×	×	PBP + AB	×
13	M	3	15.2	526	×	×	CO	×
14	G	3	15.3	472	×	×	×	AB
15	M	3	14.2	460	×	×	×	CON
16	G	3	14.3	485	×	×	×	PBP

G: gelding, M: mare, S: stallion, CO: unsupplemented control, PBP: PROBIOPlus^TM^, 30 mg/kg BW top-dressed on feed once per day; days 1–28; *n* = 8, AB: Trimethoprim/Sulfadiazine, 75 mg/kg BW by oral syringe once per day; days 1–10; *n* = 8, ×: horse not used in the trial.

**Table 2:** Shannon Evenness Index for treatments by sampling period.

Trt	Sampling Period				*p*	
	Baseline	Early	Mid	Late	trt	0.409
CO	0.6004 ± 0.005	0.6143 ± 0.007	0.5980 ± 0.005	0.5993 ± 0.005	period	0.054
AB	0.5982 ± 0.005	0.6047 ± 0.007	0.6075 ± 0.005	0.5993 ± 0.005	trt × period	0.043
PBP	0.5998 ± 0.005	0.5871 ± 0.007	0.6068 ± 0.005	0.6013 ± 0.005		
PBP + AB	0.5996 ± 0.005	0.6089 ± 0.007	0.6090 ± 0.005	0.6000 ± 0.005		

CO: unsupplemented control, AB: Trimethoprim/Sulfadiazine, 75 mg/kg BW by oral syringe once per day; days 1–10; *n* = 8, PBP: PROBIOPlus^TM^, 30 mg/kg BW top-dressed on feed once per day; days 1–28; *n* = 8.

**Table 3:** Inverse Simpson Index for treatments by sampling period.

Trt	Sampling Period				*p*	
	Baseline	Early	Mid	Late	trt	0.098
CO	10.83 ± 0.2	11.62 ± 0.4 ^a^	10.32 ± 0.2	10.33 ± 0.2	period	0.041
AB	10.32 ± 0.2	10.99 ± 0.3 ^ab^	10.78 ± 0.2	10.41 ± 0.2	trt × period	0.007
PBP	10.32 ± 0.2	9.95 ± 0.3 ^b^	10.64 ± 0.2	10.44 ± 0.2		
PBP + AB	10.60 ± 0.2	10.79 ± 0.3 ^ab^	10.87 ± 0.2	10.31 ± 0.2		

CO: unsupplemented control, AB: Trimethoprim/Sulfadiazine, 75 mg/kg BW by oral syringe once per day; days 1–10; *n* = 8, PBP: PROBIOPlus^TM^, 30 mg/kg BW top-dressed on feed once per day; days 1–28; *n* = 8. Differing superscript letters denote significantly different values.

## Data Availability

The data presented in this study are available on request from the corresponding author. The data are not publicly available due to funder intellectual property protection.

## References

[1] Gustafson R.H., Bowen R.E. (1997). Antibiotic use in animal agriculture. J. Appl. Microbiol..

[2] Khusro A., Aarti C., Buendía-Rodriguez G., Arasu M.V., Al-Dhabi N.A., Barbabosa-Pliego A. (2021). Adverse Effect of Antibiotics Administration on Horse Health: An Overview. J. Equine Vet. Sci..

[3] Gustafsson A. (2002). Antibiotic-associated diarrhoea in horses. Equine Vet. Educ..

[4] Båverud V., Gustafsson A., Franklin A., Lindholm A., Gunnarsson A. (1997). *Clostridium difficile* associated with acute colitis in mature horses treated with antibiotics. Equine Vet. J..

[5] World Health Organization Probiotics in Food: Health and Nutritional Properties and Guidelines for Evaluation. https://www.fao.org/3/a0512e/a0512e.pdf.

[6] Schoster A. (2018). Probiotic Use in Equine Gastrointestinal Disease. Vet. Clin. N. Am. Equine Pract..

[7] Quigley E.M.M. (2019). Prebiotics and Probiotics in Digestive Health. Clin. Gastroenterol. Hepatol..

[8] Vanderhoof J.A., Whitney D.B., Antonson D.L., Hanner T.L., Lupo J.V., Young R.J. (1999). *Lactobacillus GG* in the prevention of antibiotic-associated diarrhea in children. J. Pediatr..

[9] McFarland L.V., Surawicz C.M., Greenberg R.N., Elmer G.W., Moyer K.A., Melcher S.A., Bowen K.E., Cox J.L. (1995). Prevention of b-lactam-associated diarrhea by Saccharomyces boulardii compared with placebo. Am. J. Gastroenterol..

[10] Jouany J.P., Medina B., Bertin G., Julliand V. (2009). Effect of live yeast culture supplementation on hindgut microbial communities and their polysaccharidase and glycoside hydrolase activities in horses fed a high-fiber or high-starch diet. J. Anim. Sci..

[11] Swyers K.L., Burk A.O., Hartsock T.G., Ungerfeld E.M., Shelton J.L. (2008). Effects of direct-fed microbial supplementation on digestibility and fermentation end-products in horses fed low-and high-starch concentrates. J. Anim. Sci..

[12] MacNicol J.L., Renwick S., Ganobis C.M., Allen-Vercoe E., Weese J.S., Pearson W. (2023). The influence of a probiotic/prebiotic supplement on microbial and metabolic parameters of equine cecal fluid or fecal slurry in vitro. J. Anim. Sci..

[13] John J., Roediger K., Schroedl W., Aldaher N., Vervuert I. (2015). Development of intestinal microflora and occurrence of diarrhoea in sucking foals: Effects of Bacillus cereus var. toyoi supplementation. BMC Vet. Res..

[14] Walters W., Hyde E.R., Berg-Lyons D., Ackermann G., Humphrey G., Parada A., Gilbert J.A., Jansson J.K., Caporaso J.G., Fuhrman J.A. (2015). Transcribed Spacer Marker Gene Primers for Microbial Community Surveys. mSystems.

[15] Schloss P.D., Westcott S.L., Ryabin T., Hall J.R., Hartmann M., Hollister E.B., Lesniewski R.A., Oakley B.B., Parks D.H., Robinson C.J. (2009). Introducing mothur: Open-source, platform-independent, community-supported software for describing and comparing microbial communities. Appl. Environ. Microbiol..

[16] Kozich J.J., Westcott S.L., Baxter N.T., Highlander S.K., Schloss P.D. (2013). Development of a dual-index sequencing strategy and curation pipeline for analyzing amplicon sequence data on the miseq illumina sequencing platform. Appl. Environ. Microbiol..

[17] MacNicol J.L., Renwick S., Ganobis C.M., Allen-Vercoe E., Weese J.S., Pearson W. (2022). A Comparison of Methods to Maintain the Equine Cecal Microbial Environment In Vitro Utilizing Cecal and Fecal Material. Animals.

[18] Gihring T.M., Green S.J., Schadt C.W. (2012). Massively parallel rRNA gene sequencing exacerbates the potential for biased community diversity comparisons due to variable library sizes. Environ. Microbiol..

[19] Hydock K.L., Nissley S.G., Staniar W.B. (2014). A standard protocol for fecal pH measurements in the horse. Prof. Anim. Sci..

[20] Yang L., Bajinka O., Jarju P.O., Tan Y., Taal A.M., Ozdemir G. (2021). The varying effects of antibiotics on gut microbiota. AMB Express.

[21] Bajinka O., Tan Y., Abdelhalim K.A., Özdemir G., Qiu X. (2020). Extrinsic factors influencing gut microbes, the immediate consequences and restoring eubiosis. AMB Express.

[22] Lucas López R., Grande Burgos M.J., Gálvez A., Pérez Pulido R. (2017). The human gastrointestinal tract and oral microbiota in inflammatory bowel disease: A state of the science review. APMIS.

[23] Chang J.Y., Antonopoulos D.A., Kalra A., Tonelli A., Khalife W.T., Schmidt T.M., Young V.B. (2008). Decreased diversity of the fecal Microbiome in recurrent *Clostridium difficile*-associated diarrhea. J. Infect. Dis..

[24] Weese J.S., Holcombe S.J., Embertson R.M., Kurtz K.A., Roessner H.A., Jalali M., Wismer S.E. (2015). Changes in the faecal microbiota of mares precede the development of *post partum* colic. Equine Vet. J..

[25] Stewart H.L., Southwood L.L., Indugu N., Vecchiarelli B., Engiles J.B., Pitta D. (2019). Differences in the equine faecal microbiota between horses presenting to a tertiary referral hospital for colic compared with an elective surgical procedure. Equine Vet. J..

[26] Theelen M.J.P., Luiken R.E.C., Wagenaar J.A., Sloet van Oldruitenborgh-Oosterbaan M.M., Rossen J.W.A., Schaafstra F.J.W.C., van Doorn D.A., Zomer A.L. (2023). Longitudinal study of the short- and long-term effects of hospitalisation and oral trimethoprim-sulfadiazine administration on the equine faecal microbiome and resistome. Microbiome.

[27] Costa M.C., Arroyo L.G., Allen-Vercoe E., Stämpfli H.R., Kim P.T., Sturgeon A., Weese J.S. (2012). Comparison of the Fecal Microbiota of Healthy Horses and Horses with Colitis by High Throughput Sequencing of the V3-V5 Region of the 16S rRNA Gene. PLoS ONE.

[28] Costa M.C., Silva G., Ramos R.V., Staempfli H.R., Arroyo L.G., Kim P., Weese J.S. (2015). Characterization and comparison of the bacterial microbiota in different gastrointestinal tract compartments in horses. Vet. J..

[29] Salem S.E., Maddox T.W., Berg A., Antczak P., Ketley J.M., Williams N.J., Archer D.C. (2018). Variation in faecal microbiota in a group of horses managed at pasture over a 12-month period. Sci. Rep..

[30] Kennedy N.A., Walker A.W., Berry S.H., Duncan S.H., Farquarson F.M., Louis P., Thomson J.M., Satsangi J., Flint H.J., UK IBD Genetics Consortium (2014). The Impact of Different DNA Extraction Kits and Laboratories upon the Assessment of Human Gut Microbiota Composition by 16S rRNA Gene Sequencing. PLoS ONE.

[31] Lim M.Y., Park Y.S., Kim J.H., Nam Y.D. (2020). Evaluation of fecal DNA extraction protocols for human gut microbiome studies. BMC Microbiol..

[32] Wang Z., Zolnik C.P., Qiu Y., Usyk M., Wang T., Strickler H.D., Isasi C.R., Kaplan R.C., Kurland I.J., Qi Q. (2018). Comparison of Fecal Collection Methods for Microbiome and Metabolomics Studies. Front. Cell. Infect. Microbiol..

[33] Song S.J., Amir A., Metcalf J.L., Amato K.R., Xu Z.Z., Humphrey G., Knight R. (2016). Preservation Methods Differ in Fecal Microbiome Stability, Affecting Suitability for Field Studies. mSystems.

[34] Arnold C., Pilla R., Chaffin K., Lidbury J., Steiner J., Suchodolski J. (2021). Alterations in the fecal microbiome and metabolome of horses with antimicrobial-associated diarrhea compared to antibiotic-treated and non-treated healthy case controls. Animals.

[35] Moens F., de Vuyst L. (2017). Inulin-type fructan degradation capacity of *clostridium* cluster IV and XlVa butyrate- producing colon bacteria and their associated metabolic outcomes. Benef. Microbes.

[36] Guo P., Zhang K., Ma X., He P. (2020). *Clostridium* species as probiotics: Potentials and challenges. J. Anim. Sci. Biotechnol..

[37] Livanos A.E., Snider E.J., Whittier S., Chong D.H., Wang T.C., Abrams J.A., Freedberg D.E. (2018). Rapid gastrointestinal loss of Clostridial Clusters IV and XIVa in the ICU associates with an expansion of gut pathogens. PLoS ONE.

[38] Neumann A.P., McCormick C.A., Suen G. (2017). *Fibrobacter* communities in the gastrointestinal tracts of diverse hindgut-fermenting herbivores are distinct from those of the rumen. Environ. Microbiol..

[39] Holdeman L.V., Moore W.E.C. (1974). New genus, *Coprococcus*, twelve new species, and emended descriptions of four previously described species of bacteria from human feces. Int. J. Syst. Bacteriol..

[40] Nogal A., Louca P., Zhang X., Wells P.M., Steves C.J., Spector T.D., Falchi M., Valdes A.M., Menni C. (2021). Circulating Levels of the Short-Chain Fatty Acid Acetate Mediate the Effect of the Gut Microbiome on Visceral Fat. Front. Microbiol..

[41] Collinet A., Grimm P., Julliand S., Julliand V. (2021). Multidimensional Approach for Investigating the Effects of an Antibiotic–Probiotic Combination on the Equine Hindgut Ecosystem and Microbial Fibrolysis. Front. Microbiol..

[42] O’ Donnell M.M., Harris H.M., Jeffery I.B., Claesson M.J., Younge B., O’Toole P.W., Ross R.P. (2013). The core faecal bacterial microbiome of Irish Thoroughbred racehorses. Lett. Appl. Microbiol..

[43] Cai S., Li J., Hu F.Z., Zhang K., Luo Y., Janto B., Boissy R., Ehrlich G., Dong X. (2010). *Cellulosilyticum ruminicola*, a Newly Described Rumen Bacterium That Possesses Redundant Fibrolytic-Protein-Encoding Genes and Degrades Lignocellulose with Multiple Carbohydrate- Borne Fibrolytic Enzymes. Appl. Environ. Microbiol..

[44] Johnson J.S., Spakowicz D.J., Hong B.Y., Petersen L.M., Demkowicz P., Chen L., Leopold S.R., Hanson B.M., Agresta H.O., Gerstein M. (2019). Evaluation of 16S rRNA gene sequencing for species and strain-level microbiome analysis. Nat. Commun..

[45] Biddle A., Stewart L., Blanchard J., Leschine S. (2013). Untangling the Genetic Basis of Fibrolytic Specialization by Lachnospiraceae and Ruminococcaceae in Diverse Gut Communities. Diversity.

[46] Wetzel D., McBride S.M. (2020). The impact of pH on *clostridioides difficile* sporulation and physiology. Appl. Environ. Microbiol..

[47] Blackmore T.M., Dugdale A., Argo C.M., Curtis G., Pinloche E., Harris P.A., Worgan H.J., Girdwood S.E., Dougal K., Newbold C.J. (2013). Strong Stability and Host Specific Bacterial Community in Faeces of Ponies. PLoS ONE.

[48] Garber A., Hastie P., Murray J.A. (2020). Factors Influencing Equine Gut Microbiota: Current Knowledge. J. Equine Vet. Sci..

[49] Weese J.S. (2003). Evaluation of deficiencies in labeling of commercial probiotics. Can. Vet. J..

[50] Weese J.S., Martin H. (2011). Assessment of commercial probiotic bacterial contents and label accuracy. Can. Vet. J..

[51] Weese J.S. (2002). Microbiologic evaluation of commercial probiotics. J. Am. Vet. Med. Assoc..

[52] Sharma P., Tomar S.K., Goswami P., Sangwan V., Singh R. (2014). Antibiotic resistance among commercially available probiotics. Food Res. Int..

